# MiR-124a Regulates Extracellular Vesicle Release by Targeting GTPase Rabs in Lung Cancer

**DOI:** 10.3389/fonc.2020.01454

**Published:** 2020-08-20

**Authors:** Giulia Romano, Giovanni Nigita, Federica Calore, Michela Saviana, Patricia Le, Carlo M. Croce, Mario Acunzo, Patrick Nana-Sinkam

**Affiliations:** ^1^Internal Medicine, Virginia Commonwealth University, Richmond, VA, United States; ^2^Division of Pulmonary, Allergy, Critical Care and Sleep Medicine, Virginia Commonwealth University, Richmond, VA, United States; ^3^Department of Molecular Virology, Immunology and Medical Genetics, Ohio State University, Columbus, OH, United States; ^4^Department of Molecular Medicine, Sapienza University, Rome, Italy

**Keywords:** miR-124a, Rab32, Rab27, extracellular vesicles, lung cancer

## Abstract

Lung cancer is the leading cause of cancer mortality worldwide. Increased understanding of the molecular mechanisms of the disease has led to the development of novel therapies and improving outcomes. Recently, extracellular vesicles (EVs) have emerged as vehicles for the transfer of genetic information between tumors and their microenvironment and have been implicated in lung cancer initiation, progression, and response to therapy. However, the mechanisms that drive the biogenesis and selective packaging of EVs remain poorly understood. Rab family guanosine triphosphates (GTPases) and their regulators are important membrane trafficking organizers. In this study, we investigated the role of select Rab GTPases on the regulation of EV release. We found that microRNAs target Rab GTPases to regulate EV release from lung cancer cell lines. In particular, Rab32 is a target of miR-124a, and siRNA and miRNA mediated inhibition of Rab32 leads to impaired EV secretion. The downstream implications for microRNA-based regulation of EV release are currently under investigation.

## Introduction

Lung cancer remains the leading cause of cancer-related death worldwide. Despite the advent of strategies for early detection and novel therapeutics, additional efforts to curb mortality rates are still required. MicroRNAs (miRNAs) represent a group of endogenous, small, 18–25 base-long non-coding RNAs involved in many fundamental physiological processes in human development and disease. Dysregulation of miRNA expression has increasingly been associated with the initiation and progression of cancer, making miRNAs one of the most analyzed molecules in cancer research (1, 2). It is estimated that miRNAs can target ~ 20–30% of genes (3, 4). A single miRNA can target hundreds of genes, and a single gene can be regulated by many miRNAs (5). miRNAs modulate gene expression predominantly through an interaction with the 3′-untranslated region (UTR) of their target mRNAs (5). Evidence suggests that miRNAs can act as either potent oncogenes or tumor suppressor genes (2, 6–8). Extracellular vesicles (EVs) are lipid bilayer-delimited particles secreted by all cells (9, 10). EVs can be classified into two major groups depending on the biogenesis and content (9). Exosomes are believed to be in size range between 30 and 150 nm (10). EVs have been reported to contain RNA, lipids, proteins, and miRNAs released from all on of cells. An early investigation revealed that EVs tend to be more abundant in cancer cells (11, 12). More recently, it has been demonstrated that the selective release of subpopulations of EVs cancers may serve as a means for the exchange of genetic material and drive both the initiation and progression of tumors (13, 14). EVs have been implicated in necessary biological processes, including apoptosis, angiogenesis, cell cycle regulation, differentiation, and epithelial-mesenchymal transformation (15). By transferring cargo from one cell to another, EVs potently influence recipient cells' behavior that may, in turn, promote the development of a microenvironment conducive to cancer cell growth, invasion, and metastasis (12). The molecular mechanisms that drive the selective packaging and overproduction of EVs in cancer remain unclear. Recent reports suggest that there are nearly 60 different Rab GTPases in humans, making them the largest family of small GTPases (16–19). Functionally, these GTPases masterfully regulate intracellular membrane traffic (16–19) by a mechanism of acting as molecular switches that are “on” when GTP is bound and “off” when GDP is bound. Investigators have reported that Rab GTPase genes including Rab2, Rab3GAP1, Rab11FIP5, Rab27A, Rab31, Rab38, are overexpressed in cancer suggesting a potential relationship between Rab GTPase and EVs release (20, 21). In particular, it has been reported that Rab27A regulates EV release in lung cancer cell lines (22). In human glioma, it has been shown that Rab27A is regulated by miR-124a and mediates the secretion of cathepsin D (22). Furthermore, Rab27a was found to be positively correlated with EV production in a panel of 39 human-derived melanoma cell lines (23, 24). Rab32 is involved in autophagy and melanosome secretion (25–28), and inherently in collaboration with Rab38, controls post-Golgi trafficking (29). MiR-124a is epigenetically silenced in various types of cancer and plays an important role in tumor development and progression (30–34). It has been reported that miR-124a contributes to lung cancer progression and that down-regulation of miR-124a was independently associated with shorter overall and disease free survival, which suggests that miR-124a might be a clinically relevant biomarker in the treatment of lung cancer (35). MiR-124a may function as a tumor suppressor in lung cancer (36–38). MiR-124a can inhibit proliferation, glycolysis, and energy metabolism by potentially targeting the AKT1/2-glucose transporter 1/hexokinase II in non-small cell lung cancer cells (39). In a separate study, investigators demonstrated that overexpression of miR-124a suppressed NSCLC growth by targeting Akt1 and Akt2. In addition, the systemic delivery of anti-miR-124a dramatically suppressed tumorigenesis in both an NNK-induced lung cancer model and K-ras^LA1^ transgenic mice by increasing apoptosis and inhibiting cell proliferation (36). However, little is known regarding the relationship between Rabs, miR-124a, and EV release in lung cancer. Here, we investigated the relationship between miR-124a, Rab32, and their impact on EV release in lung cancer cell lines. Our study demonstrates that miR-124a regulates EV secretion through the targeting of Rab GTPase.

## Experimental Procedures

### Cell Culture and Transfections

Human lung cancer cell (PC9, A427, and H1299) lines were obtained from ATCC (Manassas, VA) and cells were maintained in RPMI-1640 medium (GIBCO, Invitrogen) supplemented with 10% FBS and 1% Penicillin/Streptomycin (Invitrogen). All cells were maintained at 5% CO2 at 37°C. All transfections were performed using lipofectamine LTX according to the manufacturer's recommendations (Thermo Fisher) for 48 h. We transfected 100 nM of miR-124a inhibitor or pre-miR-124a (Thermo Fisher). In the co-transfection experiments, we transfected 50 nM of Scr and miR-124 and 50 nM siRab27 and siRab32 (Santa Cruz).

### Cell Line EV Isolation by Ultracentrifugation

Cells were starved after growing to 70–80% confluence. After washing the cells 3X with 1X PBS RPMI serum-free medium was add. After 48 h, EVs were isolated by sequential centrifugation as described previously (40). Briefly, the medium was collected and centrifuged at 300 × g for 10 min at 4°C to pellet the cells debris then the supernatant was centrifuged at 2,000 × g for 10 min at 4°C followed by centrifugation at 10,000 × g for 30 min at 4°C. The supernatant was then ultra-centrifuged at 100,000 × g for 70 min at 4°C. The pellet was washed with PBS and ultra-centrifuged at 100,000 × g for another 70 min. The final pellet was resuspended in PBS (40).

### Nanoparticle Tracking Analysis (NanoSight)

EVs were analyzed by nanoparticle tracking, using the NanoSight NS300 system (Malvern, Great Malvern, UK). Samples were administered and recorded under controlled flow, using the NanoSight syringe pump and script control system, and for each sample, 5 videos of 60 s duration were recorded, with a 10-s delay between recordings, generating 5 replicate histograms that were averaged. Therefore, the typical number of completed tracks per sample was ~ 1,200. The area under the curve was calculated using Prism-4 software version 4.03 (Graph Pad, San Diego, CA), to give average particle counts from these replicates.

### Western Blot Analysis

Western blotting was performed on the total protein extracts from cell line samples. RIPA lyses buffer (Sigma) with protease and phosphatase inhibitor cocktails (Roche) was used for the total protein fraction. Protein were determined using the BCA protein assay (Thermo- Fisher). Thirty micrograms of total lysates were, loaded onto polyacrylamide gels, and blotted onto polyvinylidene difluoride membranes (Bio-Rad). Membranes were blocked with 5% non-fat milk in TBS 0.1% Tween-20 for 1 h and then incubated with Rabbit anti-Rab27a (1:1,000, Cell Signaling), Rabbit anti-Rab32 (1:1,000, Sigma). After washing in TBS-T, 0.1% Tween-20, the membranes were incubated with a horseradish peroxidase-conjugated goat anti-Rabbit antibody (1:2,000, Cell Signaling) and the immunoblots were visualized using ECL detection kits, (Pierce; Rockford, IL, USA). A mouse anti-β-actin antibody (1:2,000, Sigma) or, anti-GAPDH (Cell Signaling) (1:5,000) was used as the control for equal loading of total lysate.

### RNA Isolation

Total RNA for real-time quantitative PCR (qRTPCR) detection analysis was extracted from cells or tissue using TRIzol reagent according to the manufacturer's instructions (Invitrogen). RNA was cleaned with the RNA Cleanup and Concentration kit (Norgen). The RNA concentration was determined by Nanodrop.

### qRT-PCR- cDNA Synthesis

Real-time PCR was performed by using a standard TaqMan PCR Kit protocol on an Applied Biosystems 7900HT Sequence Detection System (Applied Biosystems). The 10-μL PCR included 0.67 μL of RT product, 1 μL of TaqMan Universal PCR Master Mix (Applied Biosystems), 0.2 mM TaqMan probe, 1.5 mM forward primer, and 0.7 mM reverse primer. The reactions were incubated in a 96-well plate at 95°C for 10 min, followed by 40 cycles of 95°C for 15 s and 60°C for 1 min. All reactions were run in triplicate. The threshold cycle (Ct) is defined as the fractional cycle number at which the fluorescence passes the fixed threshold. The comparative Ct method for relative quantization of gene expression (Applied Biosystems) was used to determine miRNA expression levels. The y-axis represents the 2^∧^(DCt), or the relative expression of different miRs. miR expression was calculated relative to U54 and Rab27 and Rab32 relative to GAPDH expression. Experiments were carried out in triplicate for each data point, and data analysis was performed by using software (Bio-Rad). All probes were from Applied Biosystems.

### Target Analysis

miRNA target prediction analysis between miR-124a-3p and the 3′ UTR of RAB32 was performed by using RNAhybrid tool (version 2.2) (41).

### Plasmid Construction

To generate Rab32 3′ UTR luciferase reporter constructs, the 3′ UTRs were amplified by PCR and cloned into psiCHECK2 vector at XhoI and NotI restriction sites (Promega). A mutant Rab32 3′ UTR plasmid was also constructed in the psiCHECK2 vector by deleting the miR-124a targeting sequence in the 3′ UTR region of Rab32.

### Dual-Luciferase Reporter Assay

To confirm that miR-124a can bind to the 3′ UTRs of Rab32, 2 × 10^5^ HEK293A cells were seeded in 12-well plates with overnight. Cells were then co-transfected with either WT Rab32 3′ UTR psi- CHECK2 or mutant (mt) Rab32 3′ UTR psiCHECK2 reporters and miR-124a precursors or scrambled sequence miRNA control (Thermo Fischer). Twenty four hours after co-transfection, luciferase assays were performed using the Dual-Luciferase reporter assay kit (Promega). Firefly luciferase activity was normalized to *Renilla* luciferase activity for each sample. Statistical significance was analyzed by unpaired Student's *t-*test.

### Statistical Analysis

All experiments were carried out in triplicate and repeated at least twice. Bars indicate the standard deviation of the mean. The statistical significance of the results was determined using Student's *t-*test. The data were considered significant when *p* < 0.05.

## Results

### miR-124a Targets Rab27A in Lung Cancer Cell Lines

**Figure 1:**
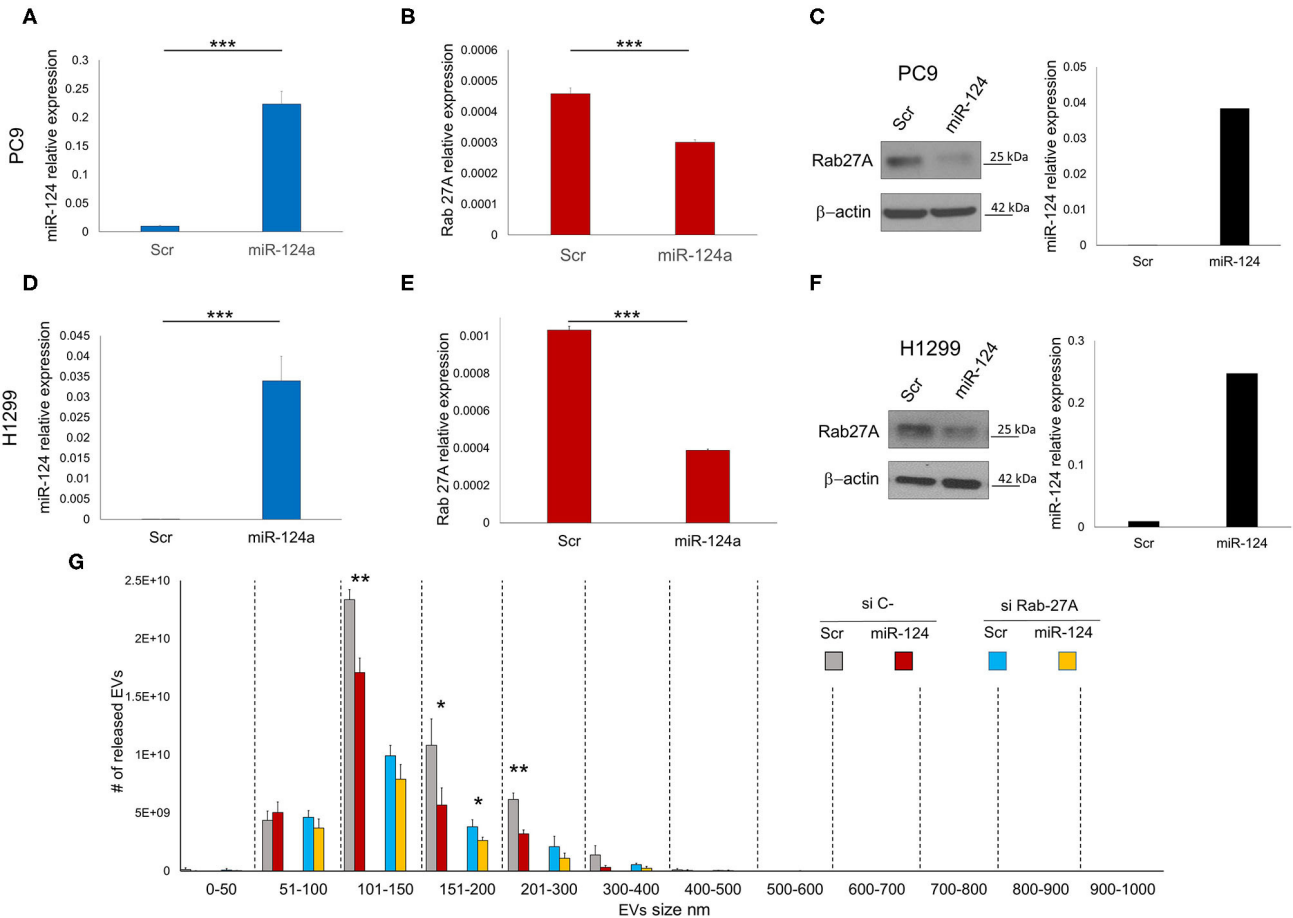
miR-124a targets Rab27A in lung cancer cell lines. **(A)** qRT-PCR showing upregulation of miR-124a after transfection of PC9 cells in triplicate **(B)** qRT-PCR showing downregulated Rab27a mRNA after miR-124a upregulation in PC9 cells in triplicate. **(C)** WB showing downregulated Rab27a protein expression (right) after miR-124a (q-RT-PCR on left) upregulation in PC9 cells. **(D)** qRT-PCR showing upregulation of miR-124a after transfection of H1299 cells **(E)** qRT-PCR showing downregulated Rab27a mRNA after miR-124a upregulation in H1299 cells. **(F)** WB showing downregulated Rab27a protein expression (right) after miR-124a (q-RT-PCR on left) upregulation in H1299 cells. **(G)** EVs number distribution divided by size after transfecting H1299 cell line with miR-124a and siRab27a. Error bars, ± s.d. *P < 0.05; **P < 0.01; ***P < 0.001.

We evaluated the effect of miR-124a overexpression in PC9 and H1299 lung cancer cell lines on Rab27a expression. MiR-124a mimic reduces Rab27a expression at both the RNA (Figures 1A,B,D,E) and protein levels (Figures 1C,F). Since Rab27a contributes to EV secretion (42, 43), and is a target of miR-124a (44, 45), we elected to investigate the role of miR-124a on Rab27a mediated EV release in lung cancer. We decreased Rab27a expression by both siRNA and miR-124a overexpression in H1299 lung cancer cell lines (Figure 1G). The distribution of EVs was quantified using NanoSight. Interestingly, miR-124a was still able to reduce 150–200 nm size range EV release in the absence of Rab27a (Figure 1G). Furthermore, in all the other groups analyzed there was still a trend in reduction of released EVs when miR-124a was co-transfected with Rab27a siRNA. We then hypothesized that miR-124a controls EV release in lung cancer through additional direct or indirect targets also taking into consideration that, as shown in Supplementary Figures 1B, 2C, the co-expression of miR-124 and siRab27 did not induce a further reduction of Rab27 at the mRNA level. The control of Rab27a and miR-124a expression levels after transfection are reported in Supplementary Figures 1A,B. We elected to not pursue the inhibition of miR-124 given that miR-124 expression in cancer is already downregulated (34) and, in general, the overexpression of a downregulated miR demonstrates a more relevant biological effect.

### miR-124a Regulates Rab32

**Figure 2:**
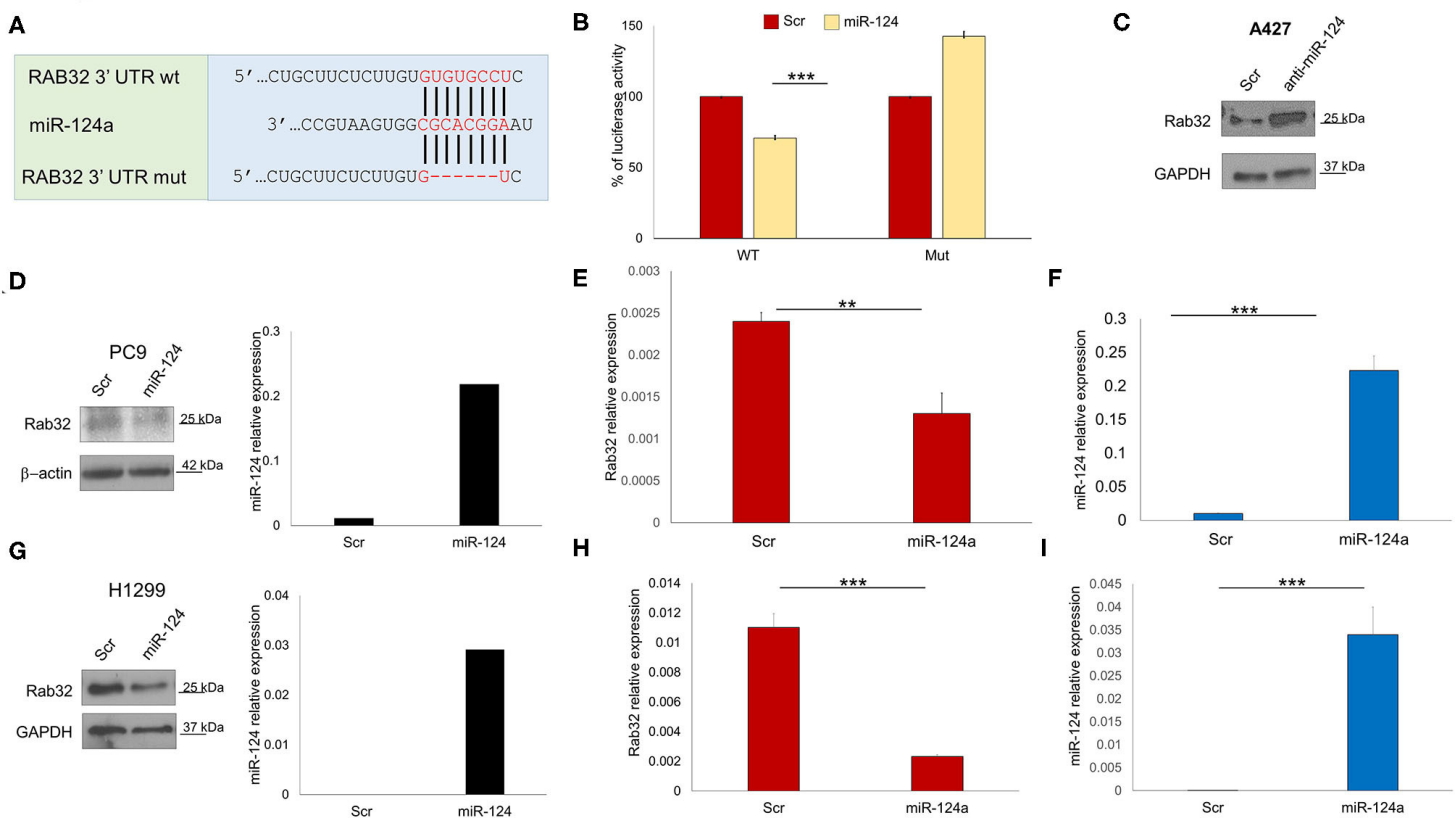
miR-124a targets Rab32 in NSCLC. **(A)** Predicted Rab32 3′ UTR binding sites for miR-124a. The alignment of the seed regions of miR-124a with Rab32 3′ UTR are shown. The sites of target deletion mutagenesis are indicated in red. **(B)** Rab32 3′ UTR is target of miR-124a. pGL3-Rab32 luciferase construct containing a WT (Left) or mutated (Right). Rab32 3′ UTR, was co-transfected with miR-124a or scrambled miRNA in 293A cells and the luciferase assay was performed. **(C)** WB showing upregulation Rab32 protein expression after miR-124a antagomir transfection in A429 cells. **(D)** WB showing downregulated Rab32 protein expression (right) after miR-124a (q-RT-PCR on left) upregulation in PC9 cells. **(E)** qRT-PCR showing downregulated Rab32a mRNA after miR-124a upregulation **(F)** in PC9 cells in triplicate. **(G)** qRT-PCR showing downregulated Rab32a mRNA after miR-124a upregulation in H1299 cells. **(H)** qRT-PCR showing downregulated Rab32a mRNA after miR-124a upregulation **(I)** in H1299 cells in triplicate. Error bars, ± s.d. **P < 0.01; ***P < 0.001.

In order to identify other possible miR-124a putative targets, we performed a bioinformatics search (RNhybrid) (41). Among the candidate targets, we selected the 3′ UTR of human Rab32 (nucleotides 858-1210, NM_006834.4), which is a protein involved in post-Golgi trafficking. Rab32 contains a region that matched the seed sequences of hsa-miR-124a (Figure 2A). To verify whether Rab32 was a direct target of miR-124a, a Rab32 3′ UTR containing miR-124a binding site was cloned into the PSICHECK2 (Promega) vector downstream the luciferase ORF. This reporter construct was used to transfect 293 cells. Overexpression of miR-124a resulted in a reduction of 3′ UTR luciferase activity (Figure 2B left). Mutation of the miR-124a binding sites within the Rab32 3′ UTR abolished the ability of miR-124a to repress the luciferase activity (Figure 2B right). Both PC9 and H1299 cells were transfected with miR-124a mimic and examined for Rab32 RNA and protein expression. We found that Rab32 was down-regulated following transfection with miR-124a mimic both at the protein (Figures 2D,G) and RNA levels (Figures 2E,F,H,I). To further validate this targeting, we transfected the A427 lung cell line with a miR-124a antagonist and examined for Rab32 protein expression. We found that Rab32 was up-regulated after transfection with the miR-124a antagonist (Figure 2C).

### Rab32 Inhibition Reduces EV Release in Lung Cancer

**Figure 3:**
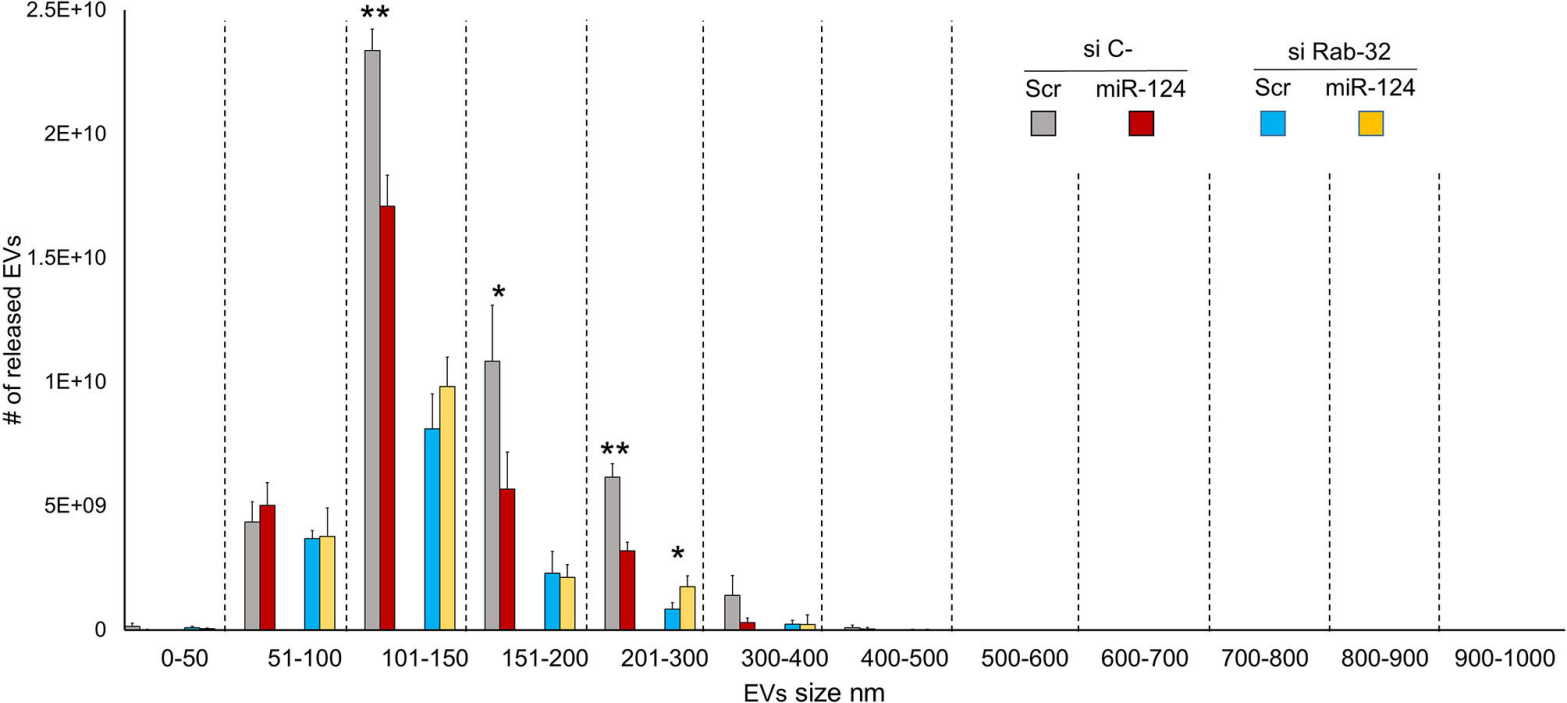
Rab32 inhibition reduces EV release in lung cancer. EVs number distribution divided by size after transfecting H1299 cell line with miR-124a and siRab32. Error bars, ± s.d. *P < 0.05; **P < 0.01.

To examine the effect of Rab32 targeting on EV release, we decreased Rab32 expression by both siRNA and miR-124a overexpression in the H1299 lung cancer cell line (Figure 3). The distribution of EVs was quantified using NanoSight. We demonstrated that in the absence of Rab32, miR-124a could no longer reduce EV release (Figure 3), and that the 200–300 nm sized EVs group actually trended in the opposite direction. Further, in this case, the co-expression of miR-124 and siRab27 did not induce a further reduction in Rab32 mRNA levels (Supplementary Figures 1B,D). The Rab32 and miR-124a expression levels after transfection are presented in Supplementary Figures 1C,D.

### Role of Rab32 and Rab27 Inhibition in EV Release in Lung Cancer

**Figure 4:**
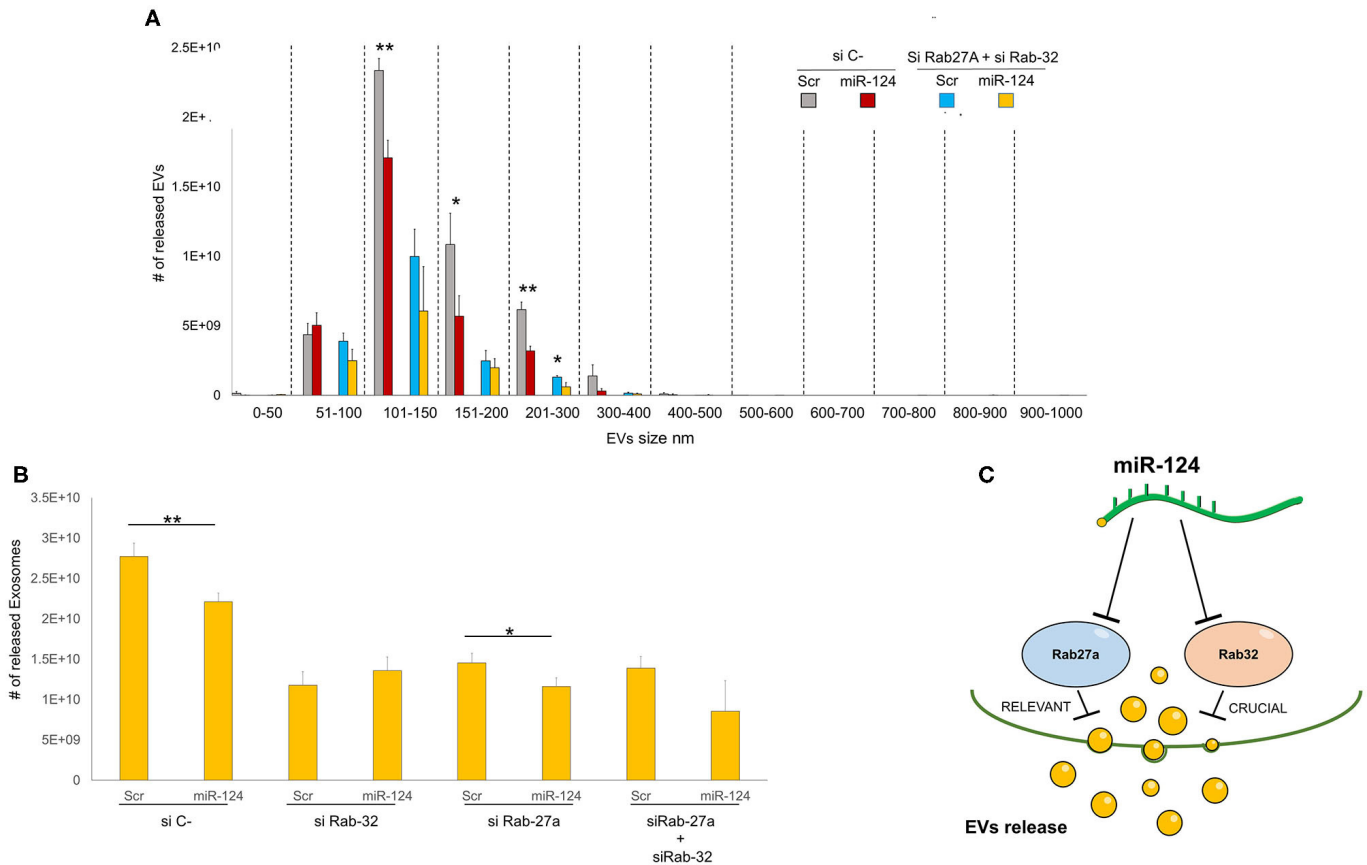
Role of Rab32 and Rab27 inhibition in EV release in lung cancer. **(A)** EVs number distribution divided by size after transfecting H1299 cell line with miR-124a, siRab32, and siRab27a **(B)** Exosome (from 50 to 150 nm group) number distribution after transfecting H1299 cell line with miR-124a, siRab32, and siRab27a **(C)** Summary diagram of our system: miR-124a targeting Rab27a and Rab32 can regulate the EV release. Rab27 results to be important in this process while Rab32 results to be crucial. Error bars, ± s.d. *P < 0.05; **P < 0.01.

We then sought to comprehensively examine the respective roles of miR-124a, Rab27a, and Rab32 on EV release in lung cancer. We combined the downregulation of both Rab-27 and Rab32 (siRNAs) with miR-124a overexpression, and then measured the total EVs and exosomes released, in the H1299 lung cancer cell line (Figures 4A,B). The distribution of EVs and exosomes was quantified using NanoSight. After down-regulating of both Rab proteins we found that miR-124a overexpression was unable repress the majority of EVs released except for those in the 200–300 nm size range. These results confirm the role of Rab32 as a target in miR-124a mediated suppression of EV release and the complementary function of Rab27a in this process. It is also noteworthy that there was a restored repressive effect of miR-124a on EV release following silencing of both Rab27a and Rab32. This observation suggests that other genes are likely to be involved in miR-124a mediated repression of EV release. Taken together, our data suggests that miR-124a functions as a crucial regulator of EV release in lung cancer. The controls of Rab27a, Rab32, and miR-124a expression are presented in Supplementary Figures 2A–C. The proposed schema by which miR-124 regulate EVs release targeting Rab27 and Rab32 is represented in Figure 4C.

### Role of siRab32 and siRab27 Inhibition on miR-124 Expression

To further investigate the connection between miR-124 and the Rabs, we evaluated miR-124 expression following siRab27 and siRab32 transfections. As shown in Supplementary Figure 3, surprisingly, the downregulation of Rab32 but not Rab27 seemed to lead to the downregulation of miR-124a. Also, the combination of siRab27, and siRab32 reduced miR-124 expression confirming the importance of the Rab32/miR-124 axis.

## Discussion

Understanding the molecular mechanisms of cancer initiation and metastasis is essential to the development of effective therapies. Cancer cells can initiate the metastatic process and targeting through EVs (14). EVs have been implicated in important biological processes, including apoptosis, angiogenesis, cell cycle regulation, differentiation, and epithelial-mesenchymal transformation (16, 20, 21). Therefore, the regulation of EV release could be leveraged for a targeted therapeutic approach (46).

In this manuscript, we investigated miR-124a as a regulator of EV release in non-small lung cancer adenocarcinoma cell lines. miR-124a is downregulated in several cancers, including lung cancer (33, 47, 48), and in a recent study, it was reported to be downregulated in 160 NSCLC cases (49). Another study suggested that the downregulation of miR-124a may be independently associated with shorter OS and DFS as well as play critical roles in the metastasis progression of lung cancer (35). Thus, investigating the function and the putative targets of miR-124a is crucial for understanding the underlying regulatory mechanisms in lung cancer.

Rab27a is a member of the RAS oncogene family of small GTPases, which contains 60 members (16) and, is involved in EV release (22). It has been shown in independent studies that miR-124a targets Rab27a (44, 45). Nevertheless, a direct role for miR-124a on EV release, through Rab27a targeting, has never been demonstrated. In this manuscript, we confirmed that miR-124a targets Rab27a in lung cancer adenocarcinoma cell lines, and we measured the impact of such targeting on EV release in terms of number and size. We found that miR-124a, when overexpressed, induces a significant reduction in EV release. To further investigate this mechanism, we suppressed Rab27a and evaluated the miR-124a effect.

Interestingly, the silencing of Rab27a proved insufficient to inhibit the repressive effect of miR-124a on EV release. Given the results obtained, we elected to investigate other miR-124a targets involved in this complex mechanism. Based on miR-124a bioinformatic prediction targeting analysis, we identified another member of the Rab family, Rab32, as a potential target for miR-124a. It has been reported that Rab32 is involved in post-Golgi trafficking (29) and was considered to be a good candidate for regulating EV release in our lung cancer model.

We first validated the direct targeting of miR-124a on Rab32 3′ UTR. We have shown that miR-124a directly targets Rab32 mRNA 3′ UTR, promoting its cellular degradation, and reducing Rab32 protein levels. To assess the biological role of Rab32 on EV release and its relationship with miR-124a in lung cancer, we silenced Rab32 and overexpressed miR-124a in a NSLC adenocarcinoma cell line similarly as we did with Rab27a. This time, differently from what we measured with Rab27a silencing, Rab32 knockdown was crucial for the observed miR-124a effect on EV release in lung cancer cell lines. Finally, we evaluated the effect of miR-124a on EVs and the subpopulation of exosomes (50–150 nm) release when Rab27a and Rab32, where both silenced. Exosomes contribute to intercellular communication in cancer through their cargo of protein and nucleic acids (50). We found that the combination of Rab27a and Rab32 silencing did not improve the inhibition of miR-124a on EV and exosome release when compared to Rab32 silencing alone. This evidence suggests that the miR-124a mediated effect on EV release is mainly dependent on Rab32. With that in consideration, shown in Supplementary Figure 3, the downregulation of Rab32 regulated the expression of endogenous miR-124. Further investigations are required to better elucidate the Rab and miR124 axis and its contribution to EV release contents and carcinogenesis.

Interestingly, Rab27a, and Rab32 co-silencing induced a partial reversal of the miR-124a effect on EV release when compared to the Rab32 silencing alone. This fact suggests that other miR-124a regulated targets may be acting on EV biogenesis and release when both Rab27a and Rab32 expression are repressed. Further investigations are required to better elucidate the role of miR-124a on EV release in lung adenocarcinoma while an independent study should be dedicated to examining the miR-124 role in other NSCLC histology like squamous cell carcinoma.

Considering the critical role of EVs in the metastatic process (24), the modulation of miR-124a expression in lung cancer may represent a therapeutic strategy for regulating lung cancer adenocarcinoma progression and metastases.

## Data Availability Statement

The raw data supporting the conclusions of this article will be made available by the authors, without undue reservation.

## Author Contributions

GR, MA, CC, and PN-S designed the study. GR, MA, GN, FC, PL, and MS designed and performed the experiments and analyzed the corresponding results. All authors contributed to the writing of the manuscript and PN-S edited it.

## Conflict of Interest

The authors declare that the research was conducted in the absence of any commercial or financial relationships that could be construed as a potential conflict of interest.

## Supplementary Material

Supplementary Figure 1 **(A)** qRT-PCR showing upregulation of miR-124a after transfection of H1299 cells as control of the Figure 1G. **(B)** qRT-PCR showing downregulation of Rab27a after transfection of H1299 cells as control of the Figure 1G. **(C)** qRT-PCR showing upregulation of miR-124a after transfection of H1299 cells as control of the Figure 3. **(D)** qRT-PCR showing downregulation of Rab32 after transfection of PC9 cells as control of the Figure 3.

Supplementary Figure 2 **(A)** qRT-PCR showing upregulation of miR-124a after transfection of H1299 cells as control of the Figure 4A. **(B)** qRT-PCR showing downregulation of Rab32 after transfection of H1299 cells as control of the Figure 4A. **(C)** qRT-PCR showing downregulation of Rab27a after transfection of H1299 cells as control of the Figure 4A.

Supplementary Figure 3 qRT-PCR showing expression of miR-124a after transfection of H1299 cells with SiRAb27, siRab32.
