# Supplementary figures and images for: MiR-124a Regulates Extracellular Vesicle Release by Targeting GTPase Rabs in Lung Cancer
Source: Front Oncol. 2020 Aug 20;10:1454. doi: 10.3389/fonc.2020.01454 (PMC7469878; doi:10.3389/fonc.2020.01454)

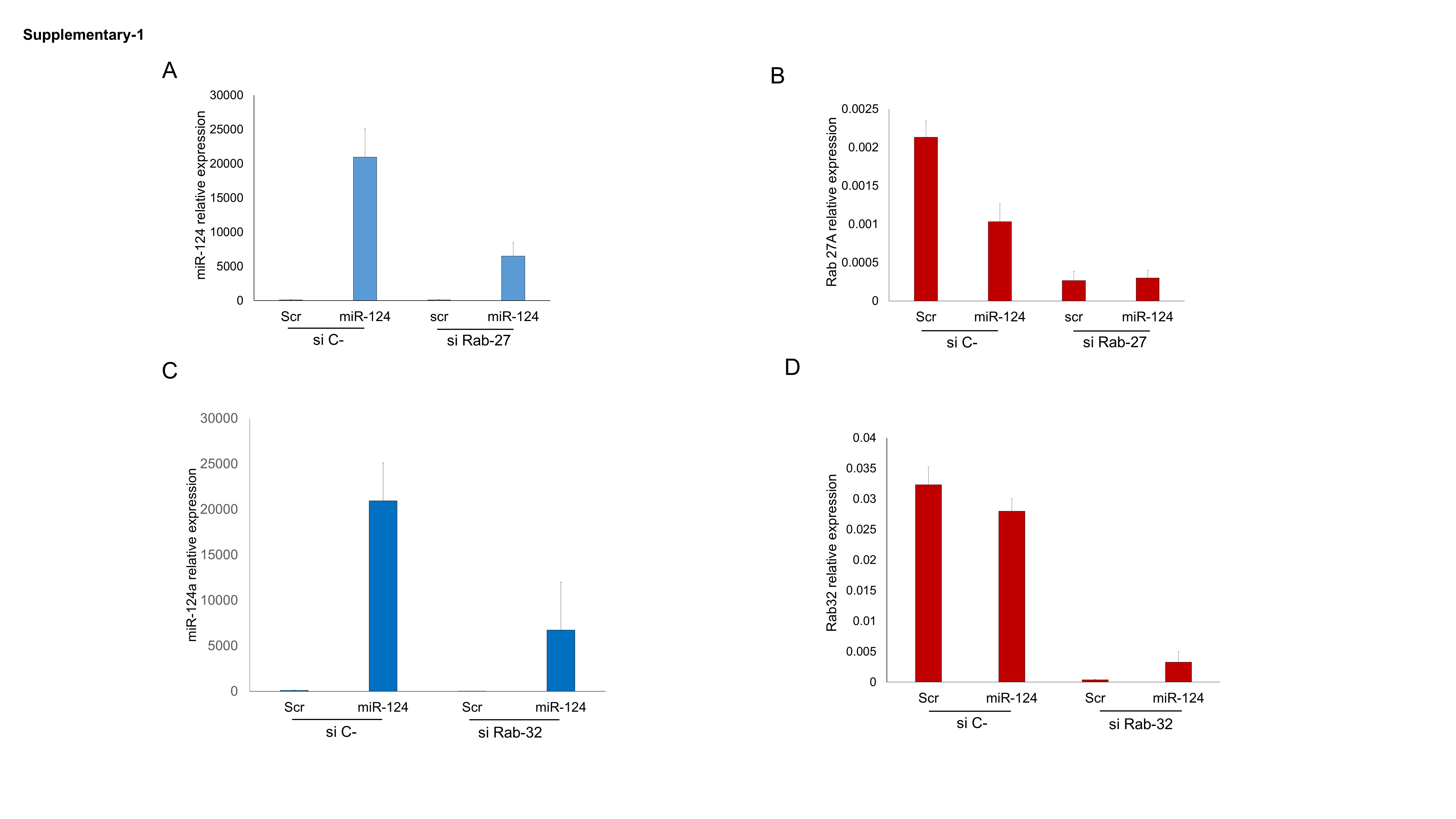

Supplement: Supplementary Figure 1 — (A) qRT-PCR showing upregulation of miR-124a after transfection of H1299 cells as control of the Figure 1G. (B) qRT-PCR showing downregulation of Rab27a after transfection of H1299 cells as control of the Figure 1G. (C) qRT-PCR showing upregulation of miR-124a after transfection of H1299 cells as control of the Figure 3. (D) qRT-PCR showing downregulation of Rab32 after transfection of PC9 cells as control of the Figure 3. [file Image_1.JPEG]

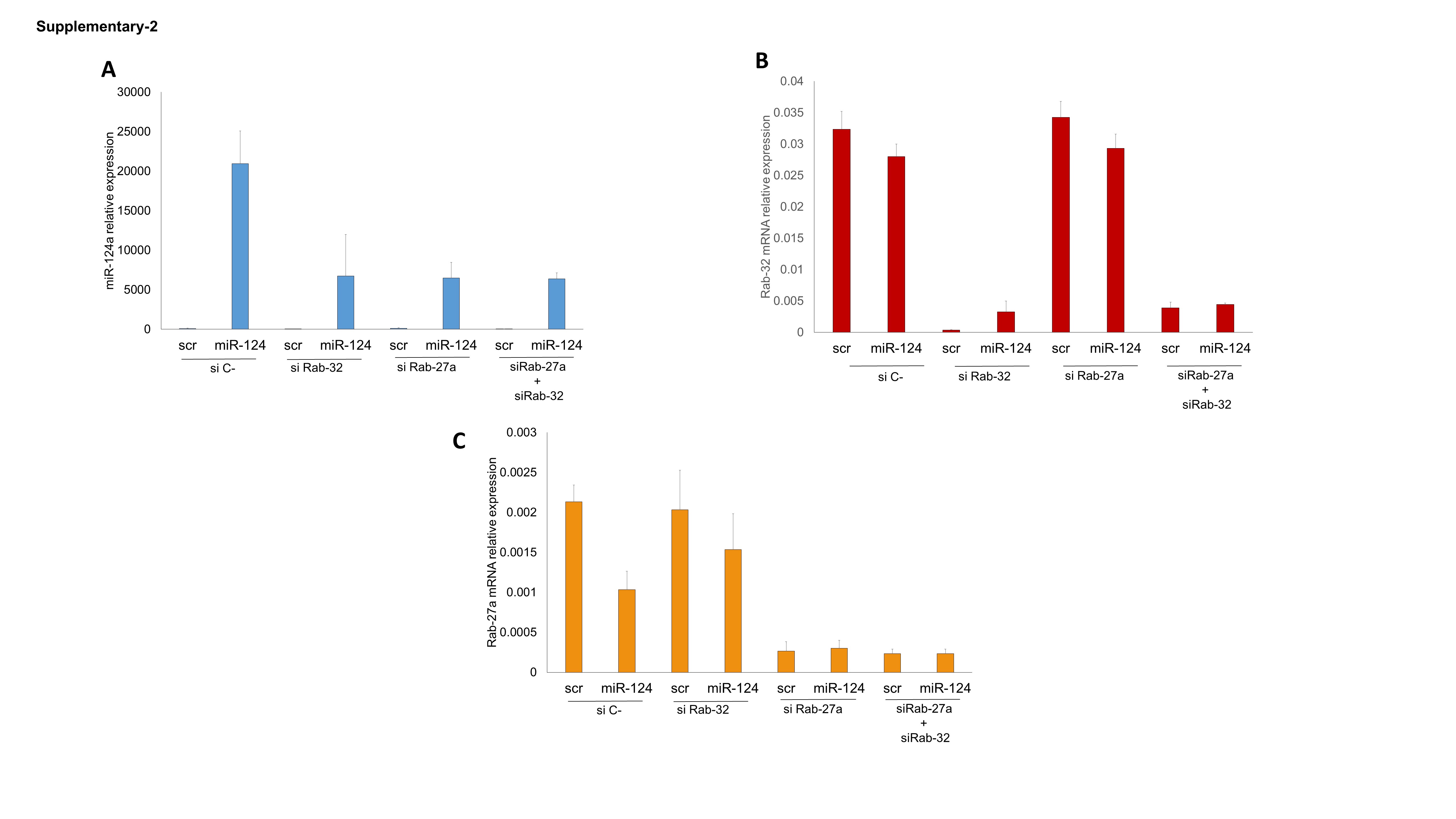

Supplement: Supplementary Figure 2 — (A) qRT-PCR showing upregulation of miR-124a after transfection of H1299 cells as control of the Figure 4A. (B) qRT-PCR showing downregulation of Rab32 after transfection of H1299 cells as control of the Figure 4A. (C) qRT-PCR showing downregulation of Rab27a after transfection of H1299 cells as control of the Figure 4A. [file Image_2.JPEG]

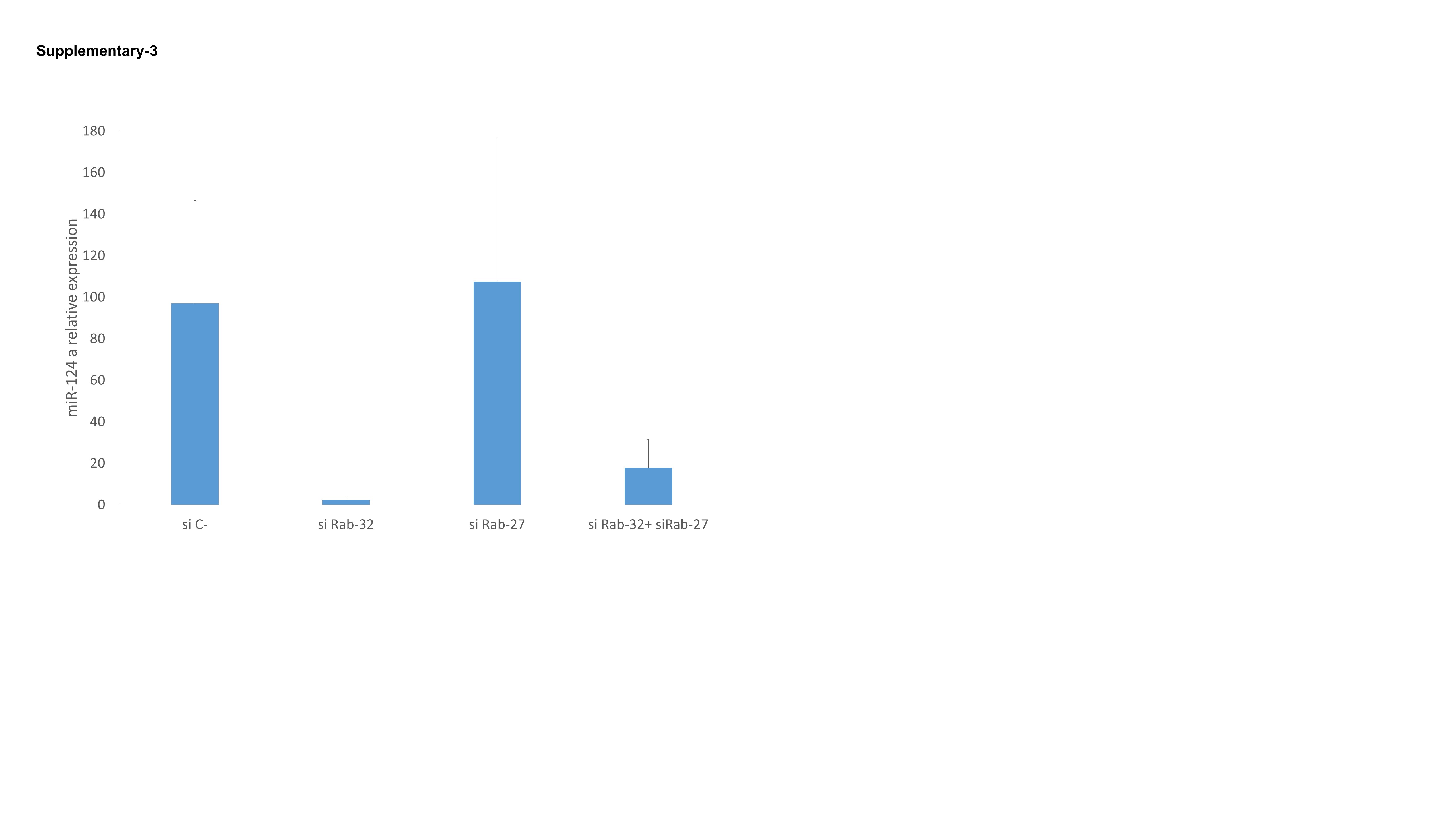

Supplement: Supplementary Figure 3 — qRT-PCR showing expression of miR-124a after transfection of H1299 cells with SiRAb27, siRab32. [file Image_3.JPEG]
